# Supplementary material for: Modelling Skylarks (Alauda arvensis) to Predict Impacts of Changes in Land Management and Policy: Development and Testing of an Agent-Based Model
Source: PLoS One. 2013 Jun 6;8(6):e65803. doi: 10.1371/journal.pone.0065803 (PMC3675089; doi:10.1371/journal.pone.0065803)
Supplement: Supporting Information S4 — The skylark ODdox as a zipped archive. (ZIP) [file pone.0065803.s004.zip › Skylark_ODdox/plants_8h.html]

ALMaSS Skylark ODdox: plants.h File Reference


ALMaSS Skylark ODdox
 2.0


- Main Page
- Related Pages
- Classes
- Files

- File List
- File Members

Classes |
Enumerations |
Variables

plants.h File Reference

`#include <vector>`  
`#include <cstdio>`  
`#include "tov_declaration.h"`  
`#include "tole_declaration.h"`

|  |  |
| --- | --- |
| Classes | |
| class | CropData |
| class | CropGrowth |

|  |  |
| --- | --- |
| Enumerations | |
| enum | Growth\_Phases {     janfirst = 0, sow, marchfirst, harvest1,     harvest2   } |

|  |  |
| --- | --- |
| Variables | |
| class CropData \* | g\_crops |
| const unsigned int | MaxNoInflections = 15 |

---

## Enumeration Type Documentation

enum Growth\_Phases

**Enumerator:**
:   |  |  |
    | --- | --- |
    | *janfirst* |  |
    | *sow* |  |
    | *marchfirst* |  |
    | *harvest1* |  |
    | *harvest2* |  |

{

janfirst = 0, sow, marchfirst, harvest1, harvest2

}

---

## Variable Documentation

class CropData\* g\_crops

Referenced by BeetleBank::BeetleBank(), VegElement::DoDevelopment(), UnsprayedFieldMargin::DoDevelopment(), FieldBoundary::FieldBoundary(), VegElement::ForceGrowthInitialize(), VegElement::ForceGrowthSpringTest(), CropRotation::GetFirstCrop(), Heath::Heath(), HedgeBank::HedgeBank(), Hedges::Hedges(), Landscape::Landscape(), Marsh::Marsh(), NaturalGrass::NaturalGrass(), Orchard::Orchard(), OrchardBand::OrchardBand(), OrchardGrass::OrchardGrass(), PermanentSetaside::PermanentSetaside(), VegElement::SetGrowthPhase(), VegElement::SetVegType(), UnsprayedFieldMargin::UnsprayedFieldMargin(), VegElement::VegElement(), YoungForest::YoungForest(), and Landscape::~Landscape().

const unsigned int MaxNoInflections = 15

Referenced by CropData::CropData(), and CropData::FindDiff().


- CJT
- MSVC
- ALMaSS Working Source
- Landscape
- plants.h
- Generated on Thu Jan 10 2013 13:15:35 for ALMaSS Skylark ODdox by
   1.8.1.1
